# Mapping of deletion breakpoints at the *CDKN2A* locus in melanoma: detection of *MTAP-ANRIL* fusion transcripts

**DOI:** 10.18632/oncotarget.7503

**Published:** 2016-02-19

**Authors:** Huaping Xie, P. Sivaramakrishna Rachakonda, Barbara Heidenreich, Eduardo Nagore, Antje Sucker, Kari Hemminki, Dirk Schadendorf, Rajiv Kumar

**Affiliations:** ^1^ Department of Gastroenterology, Tongji Hospital, Tongji Medical College, Huazhong University of Science and Technology, Wuhan, China; ^2^ Division of Molecular Genetic Epidemiology, German Cancer Research Center, Heidelberg, Germany; ^3^ Department of Dermatology, Instituto Valenciano de Oncologia, Valencia, Spain; ^4^ Department of Dermatology, University Hospital Essen, Essen, Germany; ^5^ Center for Primary Health Care Research, Lund University, Malmö, Sweden; ^6^ German Cancer Consortium (DKTK), Essen, Germany

**Keywords:** melanoma, CDKN2A, deletions, break points

## Abstract

Genomic locus at chromosome 9p21 that contains the *CDKN2A* and *CDKN2B* tumor suppressor genes is inactivated through mutations, deletions and promoter methylation in multiple human cancers. Additionally, the locus encodes an anti-sense RNA (*ANRIL*). Both hemizygous and homozygous deletions at the locus targeting multiple genes are fairly common in different cancers. We in this study investigated breakpoints in five melanoma cell lines, derived from metastasized tumors, with previously identified homozygous deletions using array comparative genomic hybridization (aCGH). For breakpoint mapping, we used primer approximation multiplex PCR (PAMP) and inverse PCR techniques. Our results showed that three cell lines carried complex rearrangements. In two other cell lines, with focal deletions of 141 kb and 181 kb, we identified fusion gene products, involving *MTAP* and *ANRIL*. We also confirmed the complex rearrangements and focal deletions in DNA from tumor tissues corresponding to three cell lines. The rapid amplification of 3′cDNA ends (3′RACE) carried out on transcripts resulted in identification of three isoforms of *MTAP-ANRIL* fusion gene. Screening of cDNA from 64 melanoma cell lines resulted in detection of fusion transcripts in 13 (20%) cell lines that involved exons 4-7 of the *MTAP* and exon 2 or 5 of the *ANRIL* genes. We also detected fusion transcripts involving *MTAP* and *ANRIL* in two of the seven primary melanoma tumors with focal deletion at the locus. The results from the study, besides identifying complex rearrangements involving *CDKN2A* locus, show frequent occurrence of fusion transcripts involving *MTAP* and *ANRIL* genes.

## INTRODUCTION

The *cyclin-dependent kinase inhibitor 2A/2B (CDKN2A/2B*) locus on chromosome 9p21, a target of frequent inactivation in various human cancers, encodes three tumor suppressors [1–6]. The p16^INK4a^ (p16 henceforth) and p14^ARF^ (ARF henceforth) are encoded from *CDKN2A* sequence, shared in alternate reading frames, and *CDKN2B* encodes p15^INK4b^ (p15 henceforth). The locus also transcribes a long intergenic noncoding RNA, termed as *antisense non-coding RNA in the INK4 locus* (*ANRIL*), involved in the repression of *CDKN2A and 2B* [7, 8]*.* Both p16 and p15 disrupt cell cycle progression through inhibition of retinoblastoma phosphorylation via disruption of CDK4/Cyclin D1 complex. ARF protein stabilizes p53 through binding to MDM2 and inhibits ubiquitin ligase activity [9, 10]. Elevated expression of any of the three proteins encoded from the *CDKN2A/2B* locus results in cell cycle arrest leading to cellular senescence [10]. Germline mutations specific to *p16* and to some extent to *ARF* impart one of the highest genetic risks of melanoma within familial settings [10–12].

Somatic inactivation of the *CDKN2A* gene, a feature common to many cancer types involves point mutations, promoter methylation or deletions at the locus [13–21]. Owing to multiple targets, deletions at 9p21 targeting *CDKN2A/B* genes constitute the predominant alterations at the locus. Previously, we reported deletions at the locus in 78% of cell lines derived from metastasized melanoma tumors [22]. The mono-allelic and bi-allelic deletions at the locus span from a few hundred kilobases to several megabases, sometimes involving the entire chromosomal arm. Deletions, besides the *CDKN2A*, also affect neighboring genes including *methylthioadenosine phosphorylase* (*MTAP*) [5, 23–25]. Despite the fact that deletions at the locus are common occurrences, the resultant breakpoints have remained mostly uncharacterized [2, 26]. The large genomic deletions that are often detected by conventional techniques such as fluorescence *in situ* hybridization (FISH), array comparative genomic hybridization (aCGH) and multiplex ligation-dependent probe amplification (MLPA) lack details about exact deletion coordinates. Therefore, a precise characterization of deletion breakpoints becomes essential for understanding the complexity of deletions at the 9p21 locus [26, 27]. In this study we used PCR based methods for identification of deletion breakpoints in melanoma cell lines followed by validation of those findings in corresponding tumors. We report chromosomal rearrangements in three cell lines and focal deletions in two cell lines. Investigation of two cell lines with focal deletions at the *CDKN2A/B* locus resulted in detection of *MTAP-ANRIL* gene fusion at deletion breakpoints*, which* we validated in corresponding tumors. Further, screening showed the presence of fusion gene transcripts in a total of 13 (20%) of 64 cell lines and two of seven primary tumors that had deletion at the locus.

## RESULTS

The pair wise copy-number data from 44 metastatic melanoma cell lines and corresponding peripheral blood mononuclear cells from a previous study was used to select cell lines with homozygous deletions (HD) [22]. 12 of the 44 cell lines carried HD at the 9p21 locus that ranged between 139 kb to 5.6 Mb (Supplementary Figure S1). Three cell lines (MaMel-30, MaMel-95, MaMel-19) with deletions less than 500 kb and two cell lines (MaMel-103a and MaMel-08a) with more than 2 Mb deletions were selected for mapping of breakpoints using primer approximation multiplex PCR (PAMP) or inverse PCR techniques (Supplementary Figure S2). The results from three cell lines, MaMel-30, MaMel-19 and MaMel-08a, were validated in the corresponding metastasized tumors; however, tumor tissues corresponding to MaMel-95 and MaMel-103a were not available.

### Breakpoint cloning with PAMP and inverse PCR

**Figure 1:**
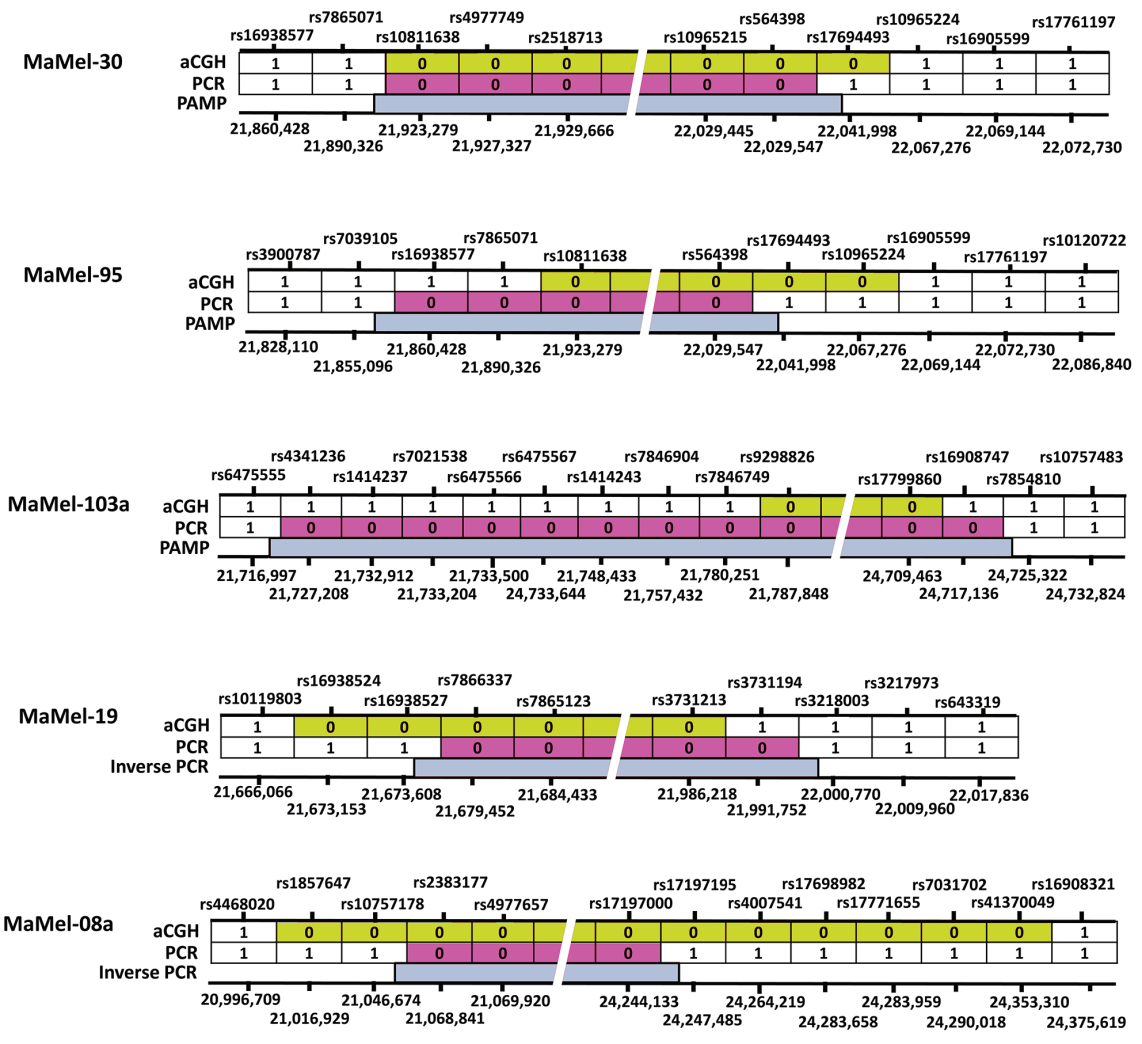
A schematic representation of homozygous deletions at 9p21 locus in 5 cell lines The SNPs marked “1” were present, detected either by aCGH or PCR. SNPs marked as “0” were deleted at the locus. The dbSNP identifications are shown on the top and respective genome coordinates (hg19) are given underneath for each cell line. The deleted regions at the loci in each cell line identified with aCGH are shown in yellow, after PCR validation in red and following exact mapping of the deletion breakpoints with PAMP/inverse PCR are indicated in blue.

The deletion breakpoints in MaMel-30, MaMel-95 and MaMel-103a were mapped using PAMP. However, due to the presence of complex rearrangements, the deletions in MaMel-19 and MaMel-08a could not be mapped with PAMP. The deletion points in MaMel-19 and MaMel-08a at telomeric and centromeric ends were independently cloned using inverse PCR. The general mapping of breakpoints was initiated by validation of single nucleotide polymorphisms (SNPs) at the deletion junctions by PCR and the aberrations in SNP calls were used to determine the correct deletion borders (Figure 1, Supplementary Table S1). The deletion coordinates described in this study are according to Human Genome Variation Society (HGVS) nomenclature version 2.0 *(last-probe-present_first-probe-deleted)_(last-probe-deleted_first-probe-present)* or *(genomic-end-position-last-positive-probe_genomic-start-position-first-negative-probe)_(genomic-end-position-last-negative-probe_genomic-start-position-first-positive-probe)* [28].

**Figure 2A–2E:**
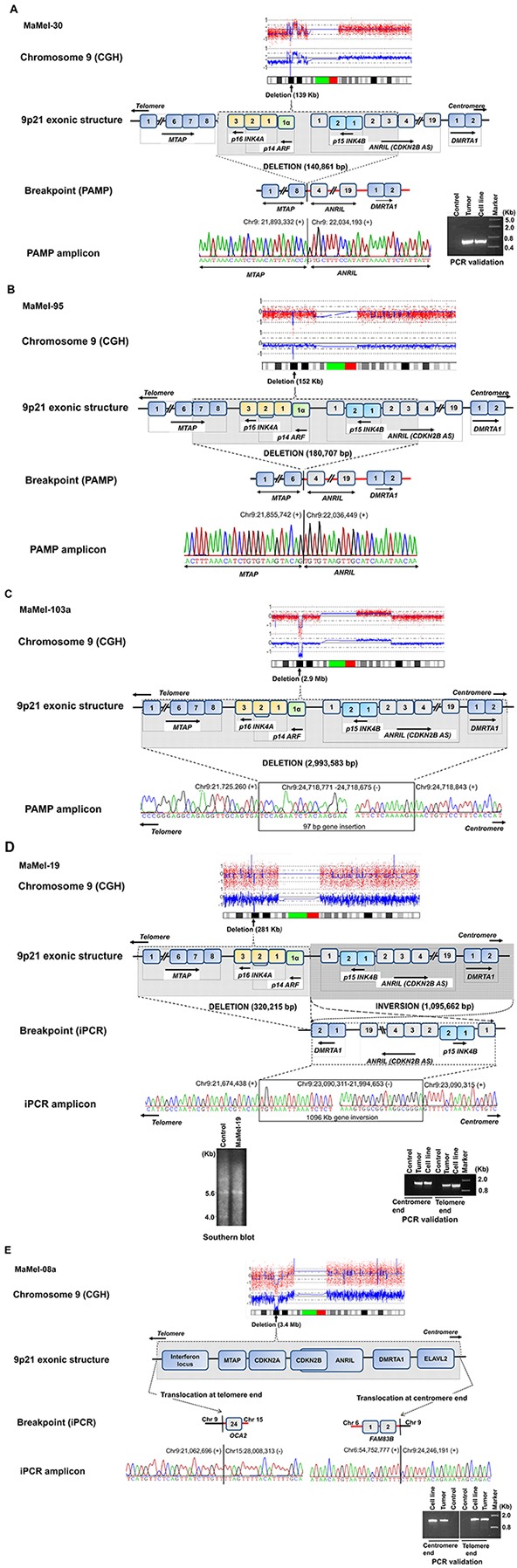
A schematic view of fine mapping of deletions at the 9p21 locus The homozygous deletions mapped at the locus by aCGH are given as molecular karyotypes followed by exonic structure and putative breakpoints. MaMel-30 cell line (2A) had a 141 kb deletion, which was confirmed in the corresponding metastasized tumor and MaMel-95 cell line (2B) had a 181 kb deletion. MaMel-103a cell line (2C) had deletion of 3.0 Mb coupled with a 97 bp insertion. MaMel-19 cell line (2D) had a deletion of 320 kb and a 1.1 Mb inversion, which was confirmed in corresponding metastasized tumor. The deletion in MaMel-19 cell line was also confirmed by Southern hybridization. MaMel-08a cell line had a deletion of 3.2 Mb and non-reciprocal translocations with chromosomes 6 and 15. Both translocations were confirmed in corresponding metastasized tumor.

In MaMel-30 cell line, the aCGH data indicated a deletion of 139 kb (Figure 1, Supplementary Table S1). Following the PCR validation, PAMP confirmed the deletion of 141 kb with coordinates *hg19 chr9:g.(21,893,332_22,034,193)del* (Supplementary Table S1). The telomeric coordinate for the deletion, chr9:21,893,332 was located within the *MTAP* gene (3′ region of Ensembl Transcript: *ENST00000380172*; intron 7 of Ensembl Transcript: *ENST00000580900*). On the centromeric side, the deletion coordinate, chr9: 22,034,193 was located within the intron 3 of *ANRIL* gene (Ensembl Transcript: *ENST00000428597*). The deletion encompassed 3′ region of *MTAP*, *CDKN2A and 2B* cluster and initial 3 exons of non-coding *ANRIL* gene (Figure 2A). The deletion was confirmed in DNA from the metastasized tumor tissue corresponding to MaMel-30 cell line by using breakpoint specific primers (Figure 2A; Supplementary Table S3).

The aCGH data for MaMel-95 indicated a deletion of 152 kb (Figure 1, Supplementary Table S1). Sequencing of the amplified product from PAMP confirmed a deletion of 181 kb with coordinates at *hg19 chr9:g.(21,855,742_22,036,449)del* (Supplementary Table S1). The sequence at the deletion junction revealed 4 bp nucleotide overlap between the fusion ends (Supplementary Table S2). The deletion on telomeric side occurred within intron 6 of the *MTAP* (Ensembl Transcript: *ENST00000380172*; intron 7 of Ensembl Transcript: *ENST00000580900*) and within intron 3 of the *ANRIL* gene (Ensembl Transcript: *ENST00000428597*), indicating a possible gene fusion between *MTAP* and *ANRIL* (Figure 2B).

In MaMel-103a cell line, the aCGH data showed a 2.9 Mb deletion (Figure 1, Supplementary Table S1). Following PCR validation, the sequencing of PAMP amplified product showed a deletion of 3.0 Mb coupled with a 97 bp insertion. The deletion and insertion coordinates were *hg19 chr9:g.(chr9:21,725,260-24,718,843)delins chr9:og. (24,718,771-24,718,675;* Figure 2C, Supplementary Table S1). At the deletion junction, a 1-2 nucleotide sequence overlap was detected between the fusion ends (Supplementary Table S2).

For MaMel-19 cell line, the aCGH indicated a deletion of 289 kb (Figure 1, Supplementary Table S1). Sequencing of the inverse PCR amplicons from both ends showed a deletion and an inversion of 320 kb and 1.1 Mb, respectively (Supplementary Table S1). The deletion included *MTAP* and *CDKN2A* genes (Chr9:21,674,438-21,994,653). The 1.1 Mb (chr9:21,994,653-23,090,311) chromosomal inversion involved *CDKN2B, ANRIL* and *DMRTA1* (Figure 2D). Southern hybridization of DNA from the cell line showed a single 5.6 kb fragment corresponding to *CDKN2B* compared to a control DNA that showed 4 kb and 5.6 kb fragments containing *CDKN2A* and *CDKN2B*, respectively (Figure 2D). The deletion and inversion in the DNA from tumor tissue corresponding to the MaMel-19 cell line was confirmmed with primers specific to the identified break points (Figure 2D, Supplementary Table S3).

The aCGH data for MaMel-08a indicated a deletion of 3.4 Mb (Figure 1, Supplementary Table S1). Inverse PCR showed non-reciprocal translocations with chromosomes 6 and 15 with a deletion of 3.2 Mb (Supplementary Table S1, Figure 2E). The deletion on the telomeric side featured a translocation involving the reverse strand of chromosome 15q12 locus (t(9;15)(p21.3;-q12)). A 4 nucleotide overlap was detected between the fusion sequences at telomeric end (Supplementary Table S2). The translocated locus corresponded to a no-gene region located 7 kb downstream of pseudogene *interferon, nu 1, pseudogene* (*IFNNP1*) on chromosome 9 (Ensembl Transcript: *ENST00000429219*) and intron 23 of *oculocutaneous albinism II* (*OCA2*) gene on chromosome 15 (Ensembl Transcript: *ENST00000354638*). On the centromeric side, the deletion accompanied a translocation of chr6p12.1 locus (t(9;6)(p21.3; p12.1)) with a 5 nucleotide overlap between the fusion sequences (Supplementary Table S2). The translocated locus corresponded to a no-gene region located 301 kb downstream of *IZUMO family member 3 (IZUMO3)* on chromosome 9 (Ensembl Transcript: *ENST00000604921*) and intron 2 of *family with sequence similarity 83, member B* (*FAM83B*)(Ensembl Transcript: *ENSG00000168143*) on chromosome 6 (Figure 2E). Using primers specific to the identified breakpoints, we confirmed the translocations on both telomeric and centromeric ends in DNA from tumor tissue corresponding to the MaMel-08a cell line (Figure 2E).

### 3′RACE of MTAP-ANRIL fusion transcript and expression in *E. coli* system

In MaMel-30 and MaMel-95 cell lines, the cloning of the breakpoints showed partial homozygous deletion of *MTAP* and *ANRIL* genes and resultant putative gene fusion. Using a combination of rapid amplification of 3′ cDNA ends (3′RACE) and *MTAP* gene specific primers, we detected two transcripts in MaMel-30 and a single transcript in MaMel-95.

One transcript detected in both cell lines had resulted from fusion of first six exons of *MTAP* gene with *ANRIL* from exon 5 onwards. In MaMel-30 cell line, the common transcript had 1467 nucleotides including 113 bp 5′untranslated region (UTR), 702 bp coding region and 652 bp 3′UTR and polyadenylation sequences (Supplementary Figure S3A). The transcript in MaMel-95 had 1425 nucleotides including 113 bp 5′UTR, 702 bp coding region and 610 bp 3′UTR and polyadenylation sequences. The sequence was identical in both the cell lines for 5′UTR and coding regions. However, two transcripts differed in length at 3′UTR. The ATG start codon was detected at position 114 and TGA stop codon was located at position 813 (Supplementary Figure S3B). The putative translation product for both transcripts encoded 233 amino acids, of which 230 amino acids were from *MTAP* and three amino acids from *ANRIL*.

The second transcript identified in MaMel-30 included first seven exons of *MTAP*, followed by *ANRIL* gene, exon 5 onwards. The transcript was 1590 bp long with 113 bp 5′UTR, 825 bp coding region and 652 bp 3′UTR and polyadenylation sequences. The ATG start codon was detected at position 114 and TGA stop codon at position 936 (Supplementary Figure S3C). The transcript putatively encoded 274 amino acids, of which 271 amino acids were from *MTAP* and three amino acids from *ANRIL* gene.

The two transcripts from ATG until the end of polyadenylation sequence were cloned into pET-20b and expressed under T7 promoter using IPTG induction. The resulting protein fractions upon detection with MTAP antibody showed corresponding protein bands for both the transcripts (data not shown).

### Screening for fusion transcripts in additional melanoma cell lines and primary melanoma tumors

**Figure 3:**
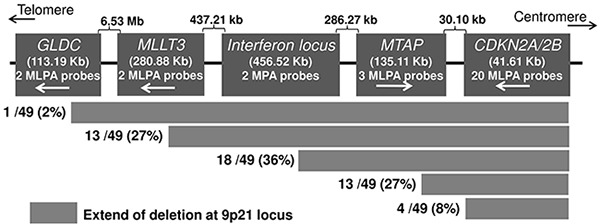
Deletion patterns at the CDKN2A/B locus detected with MLPA in melanoma cell lines The probes for the sequences further centromeric to the CDKN2A/2B locus were not available.

In order to detect the *MTAP-ANRIL* fusion gene transcript, we screened 174 metastatic melanoma cell lines for the deletion at the locus. Initial MLPA analysis identified deletions at the 9p21 locus in 100 (58%) cell lines. Out of those 100 cell lines, 49 harbored homozygous deletions and 51 had hemizygous deletions. Of the 49 cell lines with HD, 32 (65%) cell lines had the entire gene cluster at 9p21.3 locus (*MTAP-CDKN2A-CDKN2B* genes) deleted, 13 (27%) cell lines had deletions within *MTAP* or between *MTAP* and *CDKN2A*, and four (8%) cell lines had deletions within *CDKN2A* and *2B* genes (Figure 3). The *MTAP-ANRIL* fusion gene transcript was screened in cDNA from 64 cell lines that included 13 cell lines with HD within *MTAP* or between *MTAP* and *CDKN2A* and 51 cell lines with hemizygous deletions. The fusion transcript was detected in 13 (20%) cell lines, which included two cell lines, MaMel-30 and MaMel-95 that were used for initial discovery. Aligning the transcript sequences from the 13 cell lines showed in 8 (62%) fusion involved exon 6 of *MTAP* and exon 5 of *ANRIL*, 2 cell lines (15%) had gene fusion between the exon 7 of *MTAP* and exon 5 of *ANRIL*. There were solitary cases that involved - exon 5 of *MTAP* and exon 2 of *ANRIL*; exon 5 of *MTAP* and exon 5 of *ANRIL*; exon 4 of *MTAP* and exon 5 of *ANRIL*; exon 4 of *MTAP* and exon 2 of *ANRIL* (Table 1).

**Table 1:** Exonic structure of MTAP-ANRIL gene fusions observed in the melanoma cell lines / tumors analyzed

Cell line/tumor	MTAP					ANRIL						Deletion type
	Ex 4	Ex 5	Ex 6	Ex 7	Ex 8	Ex 1	Ex 2	Ex 3	Ex 4	Ex 5	Ex 6	
MaMel-30	✓	✓	✓	✓						✓	✓	HD
	✓	✓	✓							✓	✓	
MaMel-95	✓	✓	✓							✓	✓	HD
MaMel-13	✓	✓					✓		✓	✓	✓	LOH
MaMel-122	✓		✓							✓	✓	HD
UKRV-Mel-22b	✓	✓								✓	✓	LOH
Ma-Mel-66a	✓	✓	✓							✓	✓	LOH
Ma-Mel-83	✓	✓	✓	✓						✓	✓	HD
Ma-Mel-100	✓									✓	✓	LOH
Ma-Mel-24	✓	✓	✓							✓	✓	LOH
Ma-Mel-61b	✓	✓	✓							✓	✓	LOH
UKRV-Mel-20c	✓	✓	✓							✓	✓	LOH
UKRV-Mel-32	✓	✓	✓							✓	✓	HD
UKRV-Mel-22	✓						✓	✓	✓	✓	✓	HD
Tumor-1	✓	✓	✓							✓	✓	LOH
Tumor-2	✓+ intron 4										✓	LOH
	✓	✓	✓								✓	
	✓	✓	✓ + intron 6								✓	

Ex: exon number in the respective gene, HD: Homozygous deletion, LOH: Loss of heterozygosity.

The sequence data for the full length and partial transcripts have been submitted to the GenBank databases under accession numbers KT386339-KT386352.

**Figure 4:**
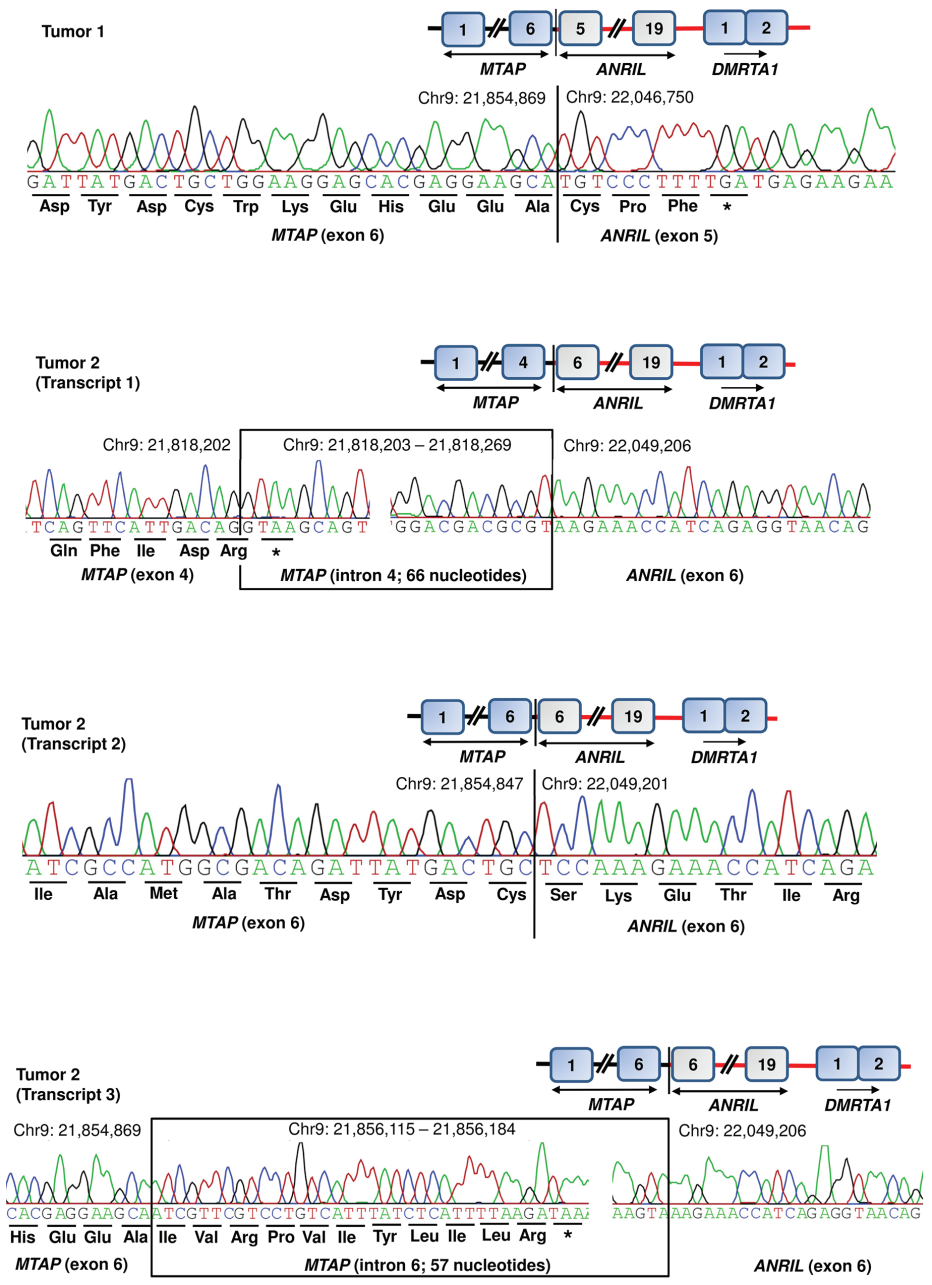
Detection of *MTAP-ANRIL* fusion transcripts in primary tumors Tumor 1 showed one and tumor 2 showed three fusion transcripts. Two of the fusion transcripts (tumor 1 and tumor 2, transcript 2) involved exon 6 of MTAP; one transcript from tumor 2 (transcript 1) involved exon 4 of MTAP together with intron 4. One transcript from tumor 2 (transcript 3) involved exon 6 of MTAP together with intron 6. Three of the four transcripts involved exon 6 of ANRIL; whereas in tumor 1, the fusion transcript included exon 5 of *ANRIL*.

Additionally, we used data from 60 primary melanoma tumors that had been previously screened for deletions at the *CDKN2A/B* locus [29]. Of 60 primary tumors, seven showed focal deletions at the *CDKN2A/B* locus that extended into *MTAP* in five tumors. Screening of cDNA from seven tumors with deletions at the *CDKN2A/B* locus showed fusion transcripts in two tumors. In one tumor, we detected fusion transcript similar to the one detected in MaMel-95 cell line involving exon 6 of *MTAP* and exon 5 of *ANRIL* with latter contributing three amino-acid residues (Figure 4, Table 1). The second tumor showed three fusion transcripts. One of those fusion transcripts (transcript 1) involved intron 4 of MTAP and exon 6 of *ANRIL*, with stop codon located within intron 4 of *MTAP* itself (Figure 4, Table 1). The second transcript (transcript 2) from the same tumor showed fusion involving exon 6 of *MTAP* and exon 6 of *ANRIL*; however, the end of the transcript remains to be determined (Figure 4, Table 1). The transcript 3 involved fusion between intron 6 of *MTAP* and exon 6 of *ANRIL* with stop codon within intron 6 of *MTAP* (Figure 4, Table 1).

## DISCUSSION

In this study we fine mapped deletions at the 9p21 locus that harbors *CDKN2A/B* complex to clone the resultant breakpoints in melanoma cell lines derived from metastasized tumors. We used a previously generated aCGH data to identify the extent of the deletions in the investigated cell lines to map the breakpoint architectures. Our results showed that in five cell lines the deletions ranged from 141 kb to 3 Mb and involved complex rearrangements including translocations, inversions, insertions and gene fusions. The availability of tumor tissues corresponding to the cell lines allowed us to confirm the breakpoints identified in cell lines through use of specific primers, which ruled out the results being cell culture artifacts. Two of the cell lines showed gene fusions involving parts of the *MTAP* and non-coding ANRIL genes resulting from the focal deletion of *CDKN2A/B* complex. Our results showed transcription of the fusion gene and *in vitro* translation. The transcription of the fusion gene involving *MTAP* and *ANRIL* as a more general phenomenon was indicated by detection of such transcripts in 13 of 64 cell lines and in two of the 7 primary melanoma tumors with focal deletion at the locus.

The cloning of breakpoints at the *CDKN2A* locus did not reveal any significant sequence homologies or an overlap at the junctions. Our observations were in accordance with previous reports in other tumors indicating that double strand breaks in 9p21 locus occur randomly and are regulated by unknown factors [5, 30]. Three cell lines with chromosomal rearrangements showed two separate deletions within the 9p chromosome, with one deletion at the *CDKN2A* locus and the other in a region approximately 700 kb centromeric to *CDKN2A*. This was evident from the sequencing data from MaMel-103a cell line that showed retention of a 97 bp fragment at the deletion junction, which appeared as an insertion. Similarly in MaMel-19 deletion at the *CDKN2A* locus accompanied an inversion of 1.1 Mb gene region. In MaMel-08a, deletions in the 9p21 locus resulted in non-reciprocal translocations with chromosome 6 on one end and with chromosome 15 on the other end of the deletion. This observation of dual deletions on chromosome 9p arm was in line with a previous study in cancer cell lines, which showed that all deletions in chromosome 9p arm depend, in some way, on deletions at *CDKN2A* locus [31].

In addition to the chromosomal rearrangements, the focal deletions at 9p21 locus resulted in fusion of parts of *MTAP* with *ANRIL*, a non-coding gene. The full length sequence of fusion transcript detected in two cell lines (MaMel-30 and MaMel-95) revealed three splice variants, two of those in MaMel-30 and one in MaMel-95; however, one transcript in MaMel-30 would encode a putative protein identical to the transcript from MaMel-95. Partial sequencing of the fusion junctions showed additional five isoforms in 11 cell lines. Recurrent observation of fusion transcripts in 13 cell lines suggested that focal deletions at the 9p21 commonly include terminal exons of *MTAP* and first five exons of *ANRIL* genes. A feature common to all fusion transcripts was skipping of exon 8 of *MTAP*. In MaMel-30 cell line, we observed that at genomic level, the deletion on the telomeric side had remained confined to the *CDKN2A* with intact *MTAP*. However, in cDNA from the same cell line we detected a fusion transcript that had skipped exon 8 of *MTAP* and had joined with *ANRIL*, exon 5 onwards.

A comparison of the fusion junction in MaMel-30 at genomic and cDNA levels indicated that the splicing in terminal exons is regulated by factors within 3′ region of *MTAP* gene [32, 33]. It can be speculated that the putative protein from *MTAP-ANRIL* fusion transcripts is analogous to truncated MTAP protein, as *ANRIL* contributed only three amino acid residues. The activity of MTAP in methionine salvage pathway is dependent on amino acid residues in exons 6 and 7; transcripts without exon 7 lack in enzymatic activity [32]. That implies that *MTAP-ANRIL* fusion transcripts without exons 7-8 detected in the present study in 11 of 13 cell lines and in two primary tumors might be enzymatically inactive. Prior to our report, a similar *MTAP-ANRIL* fusion gene was reported in a glioma tumor resulting from fusion of *MTAP* exon 4 with exon 2 of *ANRIL* and the resultant transcript contained 27 amino acids from the non-coding gene [34]. In contrast all the fusion transcripts detected in this study, contained mainly three amino acids from the *ANRIL* gene with the exception of UKRV-Mel-22 cell line that showed a transcript similar to that shown in a previous study [34]. One of the three incompletely characterized fusion transcripts in a primary tumor contained additional amino acid resides from exon 6 of the *ANRIL* gene.

The deletions at the 9p21 locus confer growth advantage to tumor cells due to occurrence of three critical tumor suppressor genes within a distance of 50 kb that regulate retinoblastoma and p53 pathways [35, 36]. Evidence from mice models and human tumors point out that *CDKN2A* represents the ‘weakest links’ of retinoblastoma and p53 pathways in melanoma development [37]. The frequency of cancer specific homozygous deletion at *CDKN2A* is higher than at any other locus within human genome [31, 36]. Cytogenetic aberrations such as translocations and gene fusions may be of greater importance in epithelial tumorigenesis [38–40]. The Cancer Genome Atlas (TCGA) Network reported 224 gene fusions in 333 cutaneous melanomas with a recurrent *GRM8-CNTNAP2* fusion in two tumors. Out of the 224 gene fusions, only one was shown to involve the fusion of *CDKN2A* locus with *NPHP4* on chr.1p36 [13]. In addition, recurrent gene fusions have been discovered in a variety of epithelial tumors, such as prostate, lung, stomach and colorectal cancers and some of the fusion genes have been confirmed to be ideal molecular targets for cancer therapy, as the *EML4-ALK* fusion gene in non-small-cell lung cancer [41–47]. Whether, the *MTAP-ANRIL* fusion transcripts detected in this study exert an oncogenic influence, if any, on tumor, remains to be determined. Nevertheless, our results in this study show that in melanoma cell lines and primary tumors with focal deletion at the *CDKN2A/B* locus, the transcription of *MTAP-ANRIL* fusion gene is a frequent occurrence.

## MATERIALS AND METHODS

### Melanoma cell lines and DNA/RNA extraction

174 cell lines included in this study were derived from metastasized tumors of 134 melanoma patients. The melanoma patients were recruited according to the eligibility criteria that included histologically confirmed melanoma of skin, mucosa or with unknown primaries. The cell lines derived from tumor biopsies were maintained in RPMI 1640 (LT) media supplemented with 10% fetal calf serum, 5 mM L-glutamine, 100 U/ml penicillin, and 100 lg/ml streptomycin at 37°C in a humidified 5% CO2 atmosphere, as described previously [22]. DNA and RNA were extracted from cell lines using Qiagen All Prep mini kit (Qiagen, Germany). Additionally, DNA samples from three tumor tissues, corresponding to three cell lines were also included in the study. The ethical approval for the study was granted by Ethics Commission of the Clinical Medicine Faculty of University of Heidelberg, Germany.

### Analysis of array-based comparative genomic hybridization (aCGH)

The aCGH was performed previously on genomic DNA from 44 cell lines and corresponding peripheral blood mononuclear cells (PBMCs) as described previously [22]. The aCGH data was taken as a basis for selection of the cell lines with HD. A copy number (CN) =0 at the position of a SNP was considered as HD and CN=1 as hemizygous deletions, CN=2 as diploid and CN>2 as amplification. Previously generated CEL files for 44 paired melanoma and blood tissues were accessible from the Gene Expression Omnibus database at NCBI (http://www.ncbi.nlm.nih.gov/geo/) under accession number GSE17534.

### Primer approximation multiplex PCR (PAMP)

Initially at the each deletion junction at least three SNPs with CN=0 and ≥1 as shown by aCGH data were validated by PCR. This validation was used to determine a minimum distance of 10-20 kb that was used for designing of primers for primer approximation multiplex PCR (PAMP). In those cases, where the distance between SNPs with CN=0 and ≥1 at the deletion border was more than 20-30 kb, we mapped additional SNPs that were present on the array. This validation was used for confirmation of the CN status determined from aCGH data.

PAMP was carried out on DNA from the selected cell lines with HD at the 9p21 locus using a modification of a previously described method [48]. A series of 9-10 forward and reverse primers (24 nt long, GC>40% and Tm>67° C) on either side of the deletion loci were designed using Primer 3 software. Specificity of the primers was determined using UCSC BLAT tool. Distance between any two forward or reverse primers was set to a maximum of 1 kb, assuming that the final amplicon will be less than 2 kb long in case primer pair nearest to the deletion junction results in amplification [48]. For mapping of the deleted locus, nine forward and nine reverse primers were used. The forward/reverse primers were split into three groups with three primers pairs in each group. Multiplex PCR was performed using all nine combinations with the forward and reverse primer sets (Supplementary Figure S5). In the final step, fresh genomic DNA was used for amplification over the breakpoints using specific primers identified in the previous step and the PCR products were subjected to Sanger sequencing (Supplementary Figure S5, Supplementary Table S3).

The PAMP reaction was carried out in a total volume of 10 μl containing 10 ng of template DNA, 0.15-0.25 μM of each forward and reverse primer, 5x HF reaction buffer, 0.11 mM of each dNTP, 0.4 U of Phusion High-fidelity DNA polymerase (New England Biolabs). Sequencing reactions were carried out using BigDye Terminator Cycle Sequencing Kit (Applied Biosystems, Warrington, UK) and run on an ABI Prism 3130XL Genetic Analyzer.

### Inverse PCR

Breakpoints in MaMel-19 and MaMel-08a cell lines were fine mapped by inverse PCR. Using the aCGH data as the basis, the breakpoints from the both the ends were mapped independently and narrowed down to approximately 1 kb by validating SNPs within and around the breakpoint. Based on the known sequence within the region, the restriction sites were determined and primers were designed in the known region adjacent to the unknown sequence. After digestion and ligation, the linear DNA was transformed into circular so that the used primers could amplify the unknown region as in a standard PCR (Supplementary Figure S4). For MaMel-19, Msp1 (New England Biolabs, Germany) and HpyCH4IV (New England Biolabs, Germany) restriction enzymes were used to digest genomic DNA for mapping of the breakpoint on telomeric and centromeric sides, respectively. For MaMel-08a, mix of SpeI, AvrII, NheI (New England Biolabs, Germany) restriction enzymes was used for the digestion. 500ng DNA was digested with corresponding restriction enzymes at 37°C for 16 hours, and the digested DNA was purified through spin columns (Fermentas, Germany) and subjected to ligation reaction with T4 DNA ligase (New England Biolabs, Germany) at 16°C for 8 h in 80 μl reaction volume. Inverse PCR was performed using LongAmpTaq 2x MasterMix (New England Biolabs, Germany) and specific primers (Supplementary Table S3). Sequencing was performed using BigDye Terminator Cycle Sequencing Kit (Applied Biosystems, Warrington, United Kingdom) and final products were run on an ABI Prism 3130XL Genetic Analyzer.

### Southern blot

Ten μg of genomic DNA extracted from cells lines using AllPrep mini kit (Qiagen, Germany) was restriction digested overnight with EcoR I (New England Biolabs, Germany) at 37°C. The digestion product was subjected to electrophoresis on a 1% agarose gel for 12 hours at 30 volt. After staining with Gel-red, the gel was soaked for 30 minutes in alkali denaturation solution (0.4N NaOH and 1M NaCl) and another 30 minutes in renaturation solution (0.5 M Tris-Cl, pH 7.2 and 1 M NaCl), then transferred onto a Hybond N+ membrane (GE-Healthcare Bio-Sciences, Germany) in 6xSCC buffer for 16-18 hours. The gel re-stained with Gel-red to confirm the complete transfer of DNA onto the membrane. The exon 2 of *CDKN2B* was PCR amplified and used as probe [49]. The probe was labeled with α-32p-dCTP using random primers and purified through column (Roche, Mannheim, Germany). The DNA immobilized membrane was prehybridized with 100 ng salmon sperm DNA for 20 minutes at 68°C in a 10 ml QuikHyb Hybridization Solution (Agilent, Germany) and then hybridized with labeled probe for 1 hour at 68°C in roller bottles. After washing twice for 15 minutes at room temperature with a 2x SSC buffer and 0.1% SDS wash solution and once for 30 minutes at 60°C with 0.1x SSC buffer and 0.1% SDS wash solution, the membrane was exposed to a photographic film overnight at −80°C.

### Rapid amplification of 3′cDNA ends (3′ RACE)

The full-length cDNA sequence of MTAP-ANRIL fusion gene was determined using 3′RACE (SMART RACE cDNA amplification kit, Clontech) and MTAP gene specific primers (Supplementary Table S3). For 3′RACE, an internal primer was designed within exon 4 of MTAP gene on the basis of results from PAMP.

The cDNA was prepared using 0.5-1 μg of total RNA using SMARTer RACE cDNA Amplification Kit (Clontech Laboratories Inc, USA). 3′RACE was performed on cDNA using internal forward primer MTAP-EX4-F (Supplementary Table S3) and Universal Primer Mix from Advantage 2 PCR Kit (Clontech Laboratories Inc, USA). The reactions were performed in 50 μl volume, with thermal conditions of 94°C for initial 2 minutes followed by 35 cycles of 94°C for 30 seconds, 68°C for 30 seconds, 72°C for 4 minutes, and final elongation time for 10 minutes. The reaction products were cloned into pCR 2.1-TOPO vectors (TOPO TA cloning kit, Invitrogen, Germany) and sequenced using vector primers. Based on the sequences, two different primers were designed specific to each fusion transcript (exon 6 of MTAP and exon 5 of *ANRIL*, F65; exon 7 of *MTAP* and exon 5 of *ANRIL*, F75; Supplementary Table S3). A nested 3′RACE was performed on the previously amplified product using fusion-specific internal primers (F65 or F75) and nested universal primer. Amplified products purified from agarose gels were cloned into pCR 2.1-TOPO vectors (TOPO TA cloning kit, Invitrogen, Germany). Finally, the full length of fusion transcripts were amplified using forward primer MTAP-EX1-F and corresponding reverse primers (Supplementary Table S3) and confirmed by DNA sequencing.

### Molecular cloning of *MTAP-ANRIL* fusion gene

A full length *MTAP-ANRIL* cDNA was ligated with linearized PCR 2.1-TOPO vector (TOPO TA cloning, Invitrogen, USA). Plasmid DNA was used as a template and the insert sequence was re-amplified using forward primer with NdeI restriction site (Fwd-primer_NdeI, Supplementary Table S3) and M13 as reverse primer. The amplicon was gel purified (PureLink Quick Gel Extraction and PCR Purification Combo Kit, Invitrogen, USA). The gel purified product and circular pET20b(+) vector were digested overnight with respective restriction enzymes (NdeI and EcoRI in MaMel95; NdeI and BamHI in MaMel30). Following the inactivation of the enzymes and column purification (MinElute Reaction Cleanup Kit, Qiagen, Germany), the digested product was incubated overnight at 16°C using T4 ligase (T4 DNA Ligase, New England Biolabs, Germany). The ligation mix was transformed into DH-5α (Z-Competent Cells, HiSS Diagnostics Germany) and plasmid DNA sequenced to confirm the position and orientation of the cloned fragment.

### Protein expression and western blot analysis

Plasmid DNA samples with pET20b-short, pET20b-long and empty pET20b vector were transformed separately into BL21(DE3) competent *E.coli* (New England Biolabs, Germany). The bacterial culture was subsequently induced for gene expression using IPTG (isopropyl b-D-1-thiogalactopyranoside) to a final concentration of 1 mM for 4 h at 37°C. The cells were harvested by centrifugation at room temperature, resuspended in 1X SDS loading dye, denatured by boiling for 3 minutes and centrifuged to remove any insoluble materials. Expression of recombinant protein was analyzed by 15% sodium dodecyl sulfate polyacrylamide gel electrophoresis (SDS—PAGE) and the protein bands were visualized with Coomassie staining solution. The approximate molecular weight was estimated by using prestained protein molecular weight standards.

The protein SDS-gels were electro-blotted onto PVDF membrane (Pierce, USA) using 64 mAmp (0.8mAmp/cm^2^ of gel) for 90 min. Membrane was blocked using 5% nonfat dry milk in TBS and 0.5% (v/v) Tween-20. Recombinant protein was detected with rabbit antihuman-MTAP (Cell Signaling, cat: 4158S; 1:1000 dilution) as primary antibody and goat anti-rabbit IR Dye 800 (1:3000 dilution; Li-Cor Biosciences) as a secondary antibody. Blots were scanned with Odyssey Infrared Imaging system (Li-Cor Biosciences, Germany), using the following conditions — Channel 700 and signal intensity of 5.

### Multiplex ligation-based probe amplification (MLPA)

Deletions at the CDKN2A locus in 174 cell lines were screened with MLPA ME024A kit (MRC, Holland). The ME024-MLPA mix contained 33 probes, out of which 23 probes were specific for the CDKN2A/2B gene (including two probes for *CDKN2B-AS1*). The *MIR31*, *MTAP* and PAX5 genes are covered by two probes each. Four more probes targeted the region between the *MIR31* gene and the 9p telomere. The probes for the sequences further centromeric to the *CDKN2A/2B* locus were not included. The kit also contained 12 reference probes, located on stable genomic regions in most tumor types. Briefly, 75-100 ng of genomic DNA was subjected to 16 h of incubation with probe mix followed by ligation reaction, followed by multiplex PCT. Fragment analysis was performed on a capillary sequencer (ABIPrism 3130xl Genetic Analyzer). The results were analyzed using Coffalyser software (MRC-Hollanf); threshold to define deletion was set at the suggested delta value of 0.3).

### Reverse transcription PCR (RT-PCR)

The reverse transcription reaction was carried out using 100 ng of total RNA, 100 pmol (0.5 μg) of Oligo(dT) primer, 4 μl of 5X reaction buffer, 0.5 μl Ribolock RNase inhibitor, 2 μl dNTP mix, 2 μl (40 U) M-MuLV reverse transcriptase in a reaction volume of 20 μl with RNAse-free water. The reaction mix was incubated at 37°C for 1 hour and heat inactivated at 70°C for 10 min. cDNA was amplified using 10pmol of gene specific forward and reverse primers (MTAP-EX4-F,ANRIL-EX6-R; Supplementary Table S3) in a 20μl reaction volume, with 1μl reverse transcription product, 2μl 10 x reaction buffer, 0.20 mM of each dNTP, 0.4U Taq DNA polymerase (Genaxxon, Germany) and 1.5 mM of MgCl_2_ under the following condition: 2 minutes at 94°C, 40 cycles of 30 seconds at 94°C, 30 seconds at 58°C, 120 seconds at 72°C, and a final extension at 72°C for 10 minutes. A nested PCR was carried out using 10 pmol of forward primer MTAP-EX4-F2 and reverse primer ANRIL-EX6-R2 (Supplementary Table S3).

### DNA sequencing

PCR products were sequenced bi-directionally with both forward and reverse primers, using Sanger chain termination method. The PCR amplicons were treated with ExoSapIT (Amersham Biosciences, Uppsala, Sweden) for 30 min at 37°C, followed by heat inactivation at 85°C for 15 min. Sequencing reaction were carried out with either forward or reverse primers, using BigDye Terminator Cycle Kit (Applied Biosystems, Warritong, UK). The reaction products after purification were analyzed on an ABI prism 3130xl Genetic analyzer (Applied Biosystems). The sequencing data were analyzed using the Sequencing Analysis 5.2 (Applied Biosystems) software.

## SUPPLEMENTARY FIGURES AND TABLES


