# Supplementary material for: Mapping of deletion breakpoints at the CDKN2A locus in melanoma: detection of MTAP-ANRIL fusion transcripts
Source: Oncotarget. 2016 Feb 19;7(13):16490–504. doi: 10.18632/oncotarget.7503 (PMC4941330; doi:10.18632/oncotarget.7503)
Supplement: Supplementary file 1 [file oncotarget-07-16490-s001.pdf]

SUPPLEMENTARY FIGURES AND TABLES

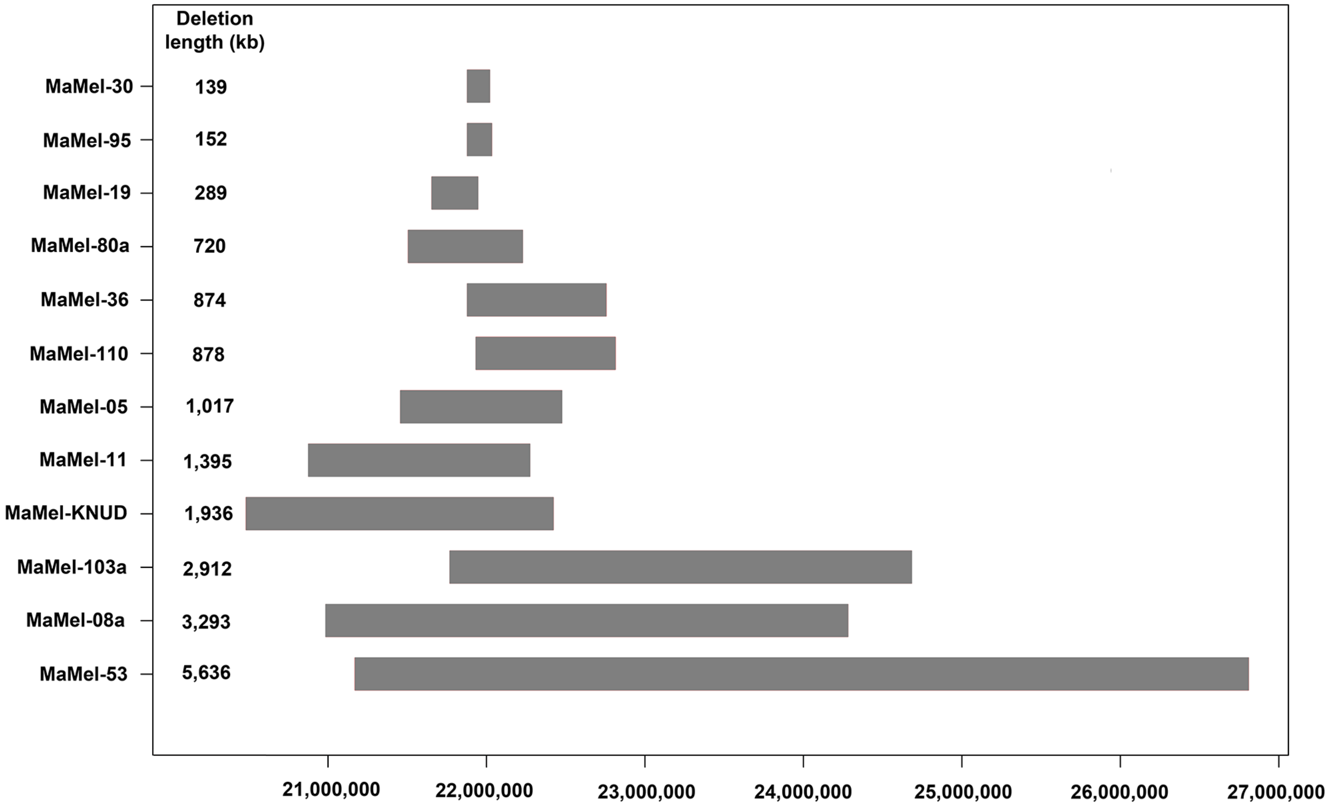

Supplementary Figure S1: A schematic representation of homozygous deletions at 9p21 locus in 12 cell lines based on aCGH data with corresponding deletion lengths.

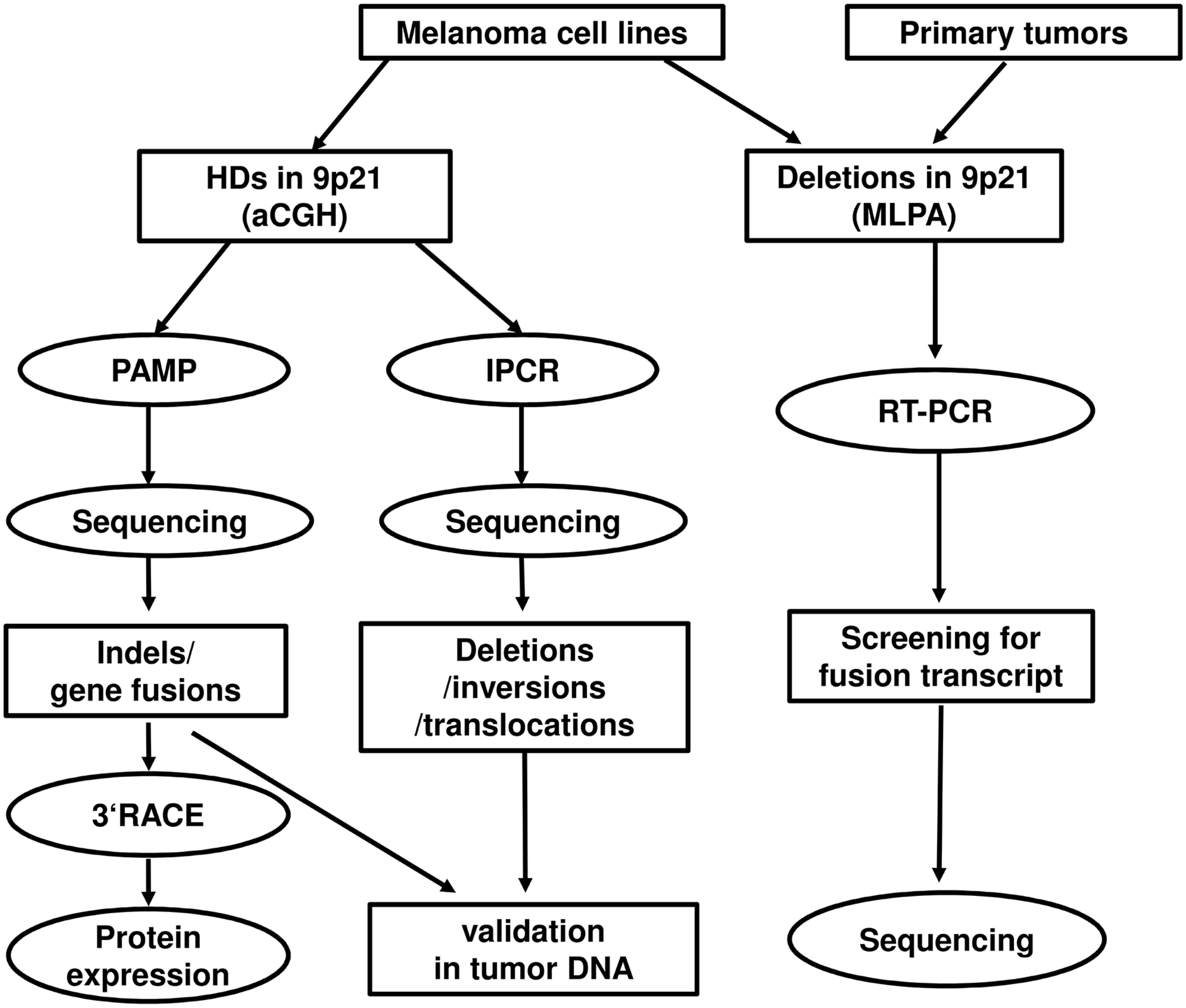

Supplementary Figure S2: A schematic flowchart of the experimental design. Deletion breakpoints based on aCGH were fine mapped in melanoma cell lines either by PAMP or inverse PCR. Breakpoints were validated in available tumor DNA corresponding to the cell lines. Transcripts corresponding to the fusion gene products were identified by Sanger sequencing followed by 3′RACE. The fusion transcripts were expressed in *E.coli* system followed by immuno detection of putative recombinant proteins. Melanoma cell lines and primary tumors were investigated by multiplex ligation probe analysis (MLPA) for deletions at the CDKN2A/B locus and cDNA from cell lines and primary tumors with deletions at the locus were screened for fusion transcripts.

A

MaMel-30 *(1-6 exons of MTAP and exon 5 of ANRIL onwards)*

```
ctccgcactgctcactcccgcgcagtgaggttggcacagccaccgctctgtggctcgcttgg
ttcccttagtcccgagcgctcgcccactgcagattcctttcccgtgcagacATGGCCTCT
                                                    M  A  S
GGCACCACCACCACCGCCGTGAAGATTGGAATAATTGGTGGAACAGGCCTGGATGATCCA
 G  T  T  T  T  A  V  K  I  G  I  I  G  G  T  G  L  D  D  P
GAAATTTTAGAAGGAAGAACTGAAAAATATGTGGATACTCCATTTGGCAAGCCATCTGAT
 E  I  L  E  G  R  T  E  K  Y  V  D  T  P  F  G  K  P  S  D
GCCTTAATTTTGGGGAAGATAAAAAATGTTGATTGCGTCCTCCTTGCAAGGCATGGAAGG
 A  L  I  L  G  K  I  K  N  V  D  C  V  L  L  A  R  H  G  R
CAGCACACCATCATGCCTTCAAAGGTCAACTACCAGGCGAACATCTGGGCTTTGAAGGAA
 Q  H  T  I  M  P  S  K  V  N  Y  Q  A  N  I  W  A  L  K  E
GAGGGCTGTACACATGTCATAGTGACCACAGCTTGTGGCTCCTTGAGGGAGGAGATTCAG
 E  G  C  T  H  V  I  V  T  T  A  C  G  S  L  R  E  E  I  Q
CCCGGCGATATTGTCATTATTGATCAGTTCATTGACAGGACCACTATGAGACCTCAGTCC
 P  G  D  I  V  I  I  D  Q  F  I  D  R  T  T  M  R  P  Q  S
TTCTATGATGGAAGTCATTCTTGTGCCAGAGGAGTGTGCCATATTCCAATGGCTGAGCCG
 F  Y  D  G  S  H  S  C  A  R  G  V  C  H  I  P  M  A  E  P
TTTTGCCCCAAAACGAGAGAGGTTCTTATAGAGACTGCTAAGAAGCTAGGACTCCGGTGC
 F  C  P  K  T  R  E  V  L  I  E  T  A  K  K  L  G  L  R  C
CACTCAAAGGGGACAATGGTCACAATCGAGGGACCTCGTTTTAGCTCCCGGGCAGAAAGC
 H  S  K  G  T  M  V  T  I  E  G  P  R  F  S  S  R  A  E  S
TTCATGTTCCGCACCTGGGGGGCGGATGTTATCAACATGACCACAGTTCCAGAGGTGGTT
 F  M  F  R  T  W  G  A  D  V  I  N  M  T  T  V  P  E  V  V
CTTGCTAAGGAGGCTGGAATTTGTTACGCAAGTATCGCCATGGCGACAGATTATGACTGC
 L  A  K  E  A  G  I  C  Y  A  S  I  A  M  A  T  D  Y  D  C
TGGAAGGAGCACGAGGAAGCATGTCCCTTTtgatgagaagaataagcctcattctgattc
 W  K  E  H  E  E  A  C  P  F  *  *
aacagcagagatcaaagaaaagacttctgttttctggccaccagatatatgttatctgtg
cttaaagaattgaaaaacacacatcaaaggagaattttcttggaaagagagggttcaagc
atcactgttaggtgtggtggaatcctttcccgagtcagtactgctttctagaagaaaacc
ggggagatctatttggaatgtatctaactccaaagaaaccatcagaggtaacaggtagga
gatatgaaacgaccttttagatatgaaccctaattgaataaaagttgccaaacaactgtt
cccaaacatctaaagaagagttttagtctaagtggaatggctggagagtatgggaagagt
tctttcctactctgtccaaacacaagcctctgtgacatttatcaaagaaatgcagccctt
taaatctgggtataagtccgaaaggtgctttccttgtgaagcttcttttgtcctctgctt
ttaggactctttctgcacactgcatcctctatgtcaccctccagtacatctgctcttaca
caaggggccccacagggactggctcacacaccagccagggcacatgggccaacttttaac
agcaaaaaaaaaaaaaaaaaaaaa
```

Supplementary Figure S3: Full length cDNA and derived amino acids sequence of *MTAP-ANRIL* fusion transcripts. The contribution to the fusion transcripts from *MTAP* is shown in black and from *ANRIL* in red **A.** MaMel-30 encoded a MTAP-ANRIL fusion transcript that included exons 1-6 of MTAP and ANRIL from exon 5 onwards. (*Continued*)

B

MaMel-95 (1-6 exons of MTAP and exon 5 of ANRIL onwards)

```
ctccgcactgctcactcccgcgcagtgaggttggcacagccaccgctctgtggctcgcttgg
ttcccttagtcccgagcgctcgcccactgcagattcctttcccgtgcagacATGGCCTCT
                                                   M  A  S
GGCACCACCACCACCGCCGTGAAGATTGGAATAATTGGTGGAACAGGCCTGGATGATCCA
 G  T  T  T  T  A  V  K  I  G  I  I  G  G  T  G  L  D  D  P
GAAATTTTAGAAGGAAGAACTGAAAAATATGTGGATACTCCATTTGGCAAGCCATCTGAT
 E  I  L  E  G  R  T  E  K  Y  V  D  T  P  F  G  K  P  S  D
GCCTTAATTTTGGGGAAGATAAAAAATGTTGATTGCGTCCTCCTTGCAAGGCATGGAAGG
 A  L  I  L  G  K  I  K  N  V  D  C  V  L  L  A  R  H  G  R
CAGCACACCATCATGCCTTCAAAGGTCAACTACCAGGCGAACATCTGGGCTTTGAAGGAA
 Q  H  T  I  M  P  S  K  V  N  Y  Q  A  N  I  W  A  L  K  E
GAGGGCTGTACACATGTCATAGTGACCACAGCTTGTGGCTCCTTGAGGGAGGAGATTCAG
 E  G  C  T  H  V  I  V  T  T  A  C  G  S  L  R  E  E  I  Q
CCCGGCGATATTGTCATTATTGATCAGTTCATTGACAGGACCACTATGAGACCTCAGTCC
 P  G  D  I  V  I  I  D  Q  F  I  D  R  T  T  M  R  P  Q  S
TTCTATGATGGAAGTCATTCTTGTGCCAGAGGAGTGTGCCATATTCCAATGGCTGAGCCG
 F  Y  D  G  S  H  S  C  A  R  G  V  C  H  I  P  M  A  E  P
TTTAGCCCCAAAACGAGAGAGGTTCTTATAGAGACTGCTAAGAAGCTAGGACTCCGGTGC
 F  S  P  K  T  R  E  V  L  I  E  T  A  K  K  L  G  L  R  C
CACTCAAAGGGGACAATGGTCACAATCGAGGGACCTCGTTTTAGCTCCCGGGCAGAAAGC
 H  S  K  G  T  M  V  T  I  E  G  P  R  F  S  S  R  A  E  S
TTCATGTTCCGCACCTGGGGGGCGGATGTTATCAACATGACCACAGTTCCAGAGGTGGTT
 F  M  F  R  T  W  G  A  D  V  I  N  M  T  T  V  P  E  V  V
CTTGCTAAGGAGGCTGGAATTTGTTACGCAAGTATCGCCATGGCGACAGATTATGACTGC
 L  A  K  E  A  G  I  C  Y  A  S  I  A  M  A  T  D  Y  D  C
TGGAAGGAGCACGAGGAAGCATGTCCCTTTtgatgagaagaataagcctcattctgattc
 W  K  E  H  E  E  A  C  P  F  *  *
aacagcagagatcaaagaaaagacttctgttttctggccaccagatatatgttatctgtg
cttaaagaattgaaaaacacacatcaaaggagaattttcttggaaagagagggttcaagc
atcactgttaggtgtggtggaatcctttcccgagtcagtactgctttctaggagaaaacc
ggggagatctatttggaatgtatctaactccaaagaaaccatcagaggtaacagccagag
ctaccttccagatgacttctttctaccactttctttcttcccagtgtaagagaatgcaag
tatatgctgatgtttggagcaagaacgttcaaaaattttcttattaacgtaacttctaat
ggaaatacagtatactactatggtgcatacaaagaagaaatagcaacatatatttgtttt
agacctgattgctgtattttattccttaggtcctccttgatgtcttgtagaccgtcacta
gctaattgttacagccatcgaaggaaaaaataaggatttttacttaggacatacgtaaca
gaaaagagatggtttagcaaaaaaaaaaaaaaaaaaaaaaaaa
```

Supplementary Figure S3: (*Continued*) Full length cDNA and derived amino acids sequence of *MTAP-ANRIL* fusion transcripts. B. MaMel-95 encoded a MTAP-ANRIL fusion transcript that included exons 1-6 of MTAP and ANRIL, exon 5 onwards. (*Continued*)

C

MaMel-30 *(1-7 exons of MTAP and exon 5 of ANRIL onwards)*

```
ctccgcactgctcactcccgcgcagtgaggttggcacagccaccgctctgtggctcgcttgg
ttcccttagtcccgagcgctcgcccactgcagattcctttcccgtgcagacATGGCCTCT
                                                     M  A  S
GGCACCACCACCACCGCCGTGAAGATTGGAATAATTGGTGGAACAGGCCTGGATGATCCA
 G  T  T  T  T  A  V  K  I  G  I  I  G  G  T  G  L  D  D  P
GAAATTTTAGAAGGAAGAACTGAAAAATATGTGGATACTCCATTTGGCAAGCCATCTGAT
 E  I  L  E  G  R  T  E  K  Y  V  D  T  P  F  G  K  P  S  D
GCCTTAATTTTGGGGAAGATAAAAAATGTTGATTGCGTCCTCCTTGCAAGGCATGGAAGG
 A  L  I  L  G  K  I  K  N  V  D  C  V  L  L  A  R  H  G  R
CAGCACACCATCATGCCTTCAAAGGTCAACTACCAGGCGAACATCTGGGCTTTGAAGGAA
 Q  H  T  I  M  P  S  K  V  N  Y  Q  A  N  I  W  A  L  K  E
GAGGGCTGTACACATGTCATAGTGACCACAGCTTGTGGCTCCTTGAGGGAGGAGATTCAG
 E  G  C  T  H  V  I  V  T  T  A  C  G  S  L  R  E  E  I  Q
CCCGGCGATATTGTCATTATTGATCAGTTCATTGACAGGACCACTATGAGACCTCAGTCC
 P  G  D  I  V  I  I  D  Q  F  I  D  R  T  T  M  R  P  Q  S
TTCTATGATGGAAGTCATTCTTGTGCCAGAGGAGTGTGCCATATTCCAATGGCTGAGCCG
 F  Y  D  G  S  H  S  C  A  R  G  V  C  H  I  P  M  A  E  P
TTTTGCCCCAAAACGAGAGAGGTTCTTATAGAGACTGCTAAGAAGCTAGGACTCCGGTGC
 F  C  P  K  T  R  E  V  L  I  E  T  A  K  K  L  G  L  R  C
CACTCAAAGGGGACAATGGTCACAATCGAGGGACCTCGTTTTAGCTCCCGGGCAGAAAGC
 H  S  K  G  T  M  V  T  I  E  G  P  R  F  S  S  R  A  E  S
TTCATGTTCCGCACCTGGGGGGCGGATGTTATCAACATGACCACAGTTCCAGAGGTGGTT
 F  M  F  R  T  W  G  A  D  V  I  N  M  T  T  V  P  E  V  V
CTTGCTAAGGAGGCTGGAATTTGTTACGCAAGTATCGCCATGGCGACAGATTATGACTGC
 L  A  K  E  A  G  I  C  Y  A  S  I  A  M  A  T  D  Y  D  C
TGGAAGGAGCACGAGGAAGCAGTTTCGGTGGACCGGGTCTTAAAGACCCTGAAAGAAAAC
 W  K  E  H  E  E  A  V  S  V  D  R  V  L  K  T  L  K  E  N
GCTAATAAAGCCAAAAGCTTACTGCTCACTACCATACCTCAGATAGGGTCCACAGAATGG
 A  N  K  A  K  S  L  L  L  T  T  I  P  Q  I  G  S  T  E  W
TCAGAAACCCTCCATAACCTGAAGTGTCCCTTTTGATGAGAAGAATAAGCCTCATTCTGA
 S  E  T  L  H  N  L  K  C  P  F  *  *
ttcaacagcagagatcaaagaaaagacttctgttttctggccaccagatatatgttatct
gtgcttaaagaattgaaaaacacacatcaaaggagaattttcttggaaagagagggttca
agcatcactgttaggtgtggtggaatcctttcccgagtcagtactgctttctagaagaaa
accggggagatctatttggaatgtatctaactccaaagaaaccatcagaggtaacaggta
ggagatatgaaacgaccttttagatatgaaccctaattgaataaaagttgccaaacaact
gttcccaaacatctaaagaagagttttagtctaagtggaatggctggagagtatgggaag
agttctttcctactctgtccaaacacaagcctctgtgacatttatcaaagaaatgcagcc
ctttaaatctgggtataagtccgaaaggtgctttccttgtgaagcttcttttgtcctctg
cttttaggactctttctgcacactgcatcctctatgtcaccctccagtacatctgctctt
acacaaggggccccacagggactggctcacacaccagccagggcacatgggccaactttt
aacagcaaaaaaaaaaaaaaaaaaaaaa
```

Supplementary Figure S3: (*Continued*) Full length cDNA and derived amino acids sequence of *MTAP-ANRIL* fusion transcripts. C. MaMel-30 also showed a second MTAP-ANRIL fusion transcript that included exons 1-7 of MTAP and ANRIL exon 5 onwards. The sequence data have been submitted to the GenBank databases under accession numbers KT386339-KT386341.

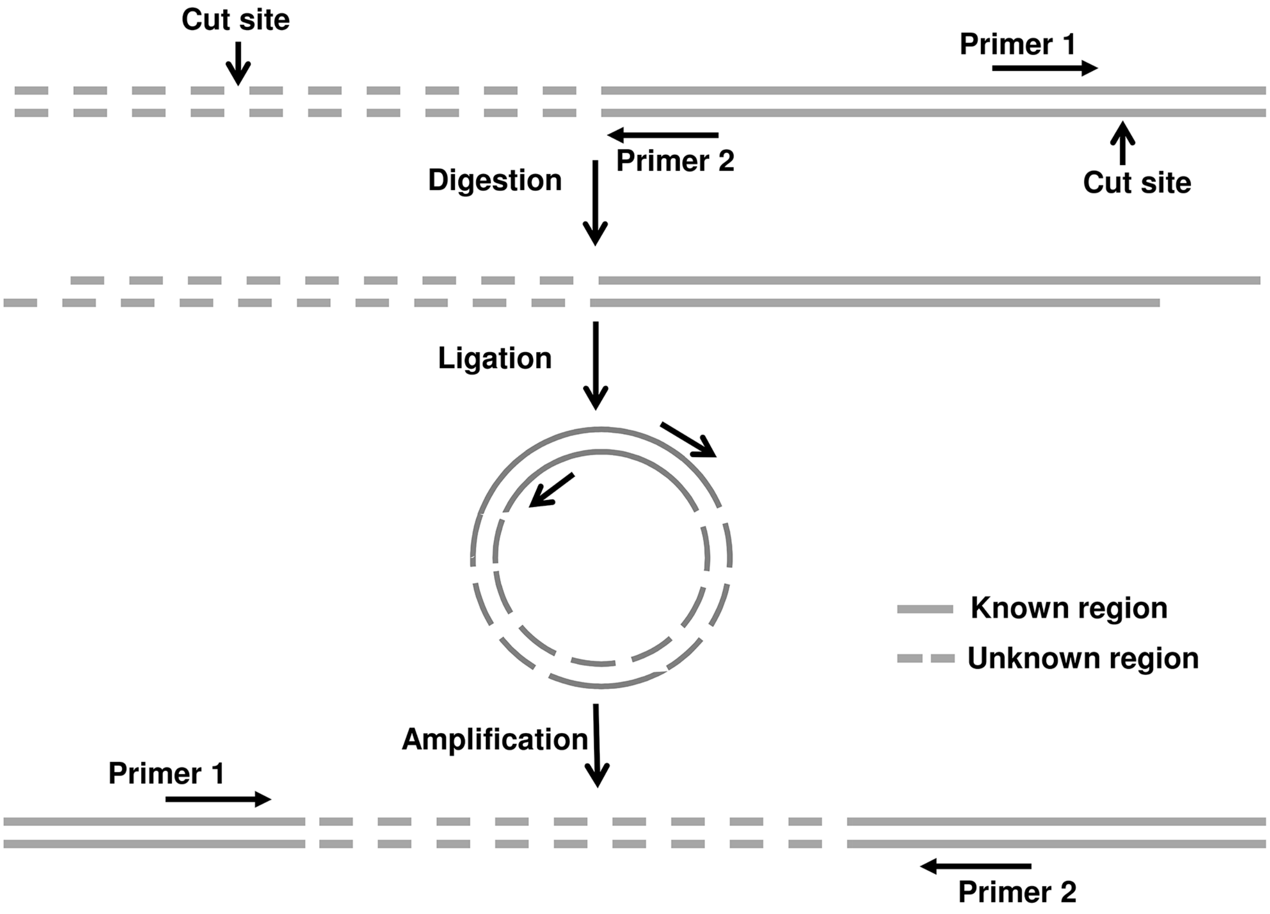

Supplementary Figure S4: A schematic representation of inverse PCR methodology. A primer pair was designed in the known sequence of DNA adjacent to the unknown region. A rare cutting restriction enzyme was selected within the known part of the sequence and digested to generate a template that was self-ligated to form a circular DNA. PCR was performed using forward and reverse primers that amplify the circular template. The amplicons were sequenced to determine the unknown DNA sequence.

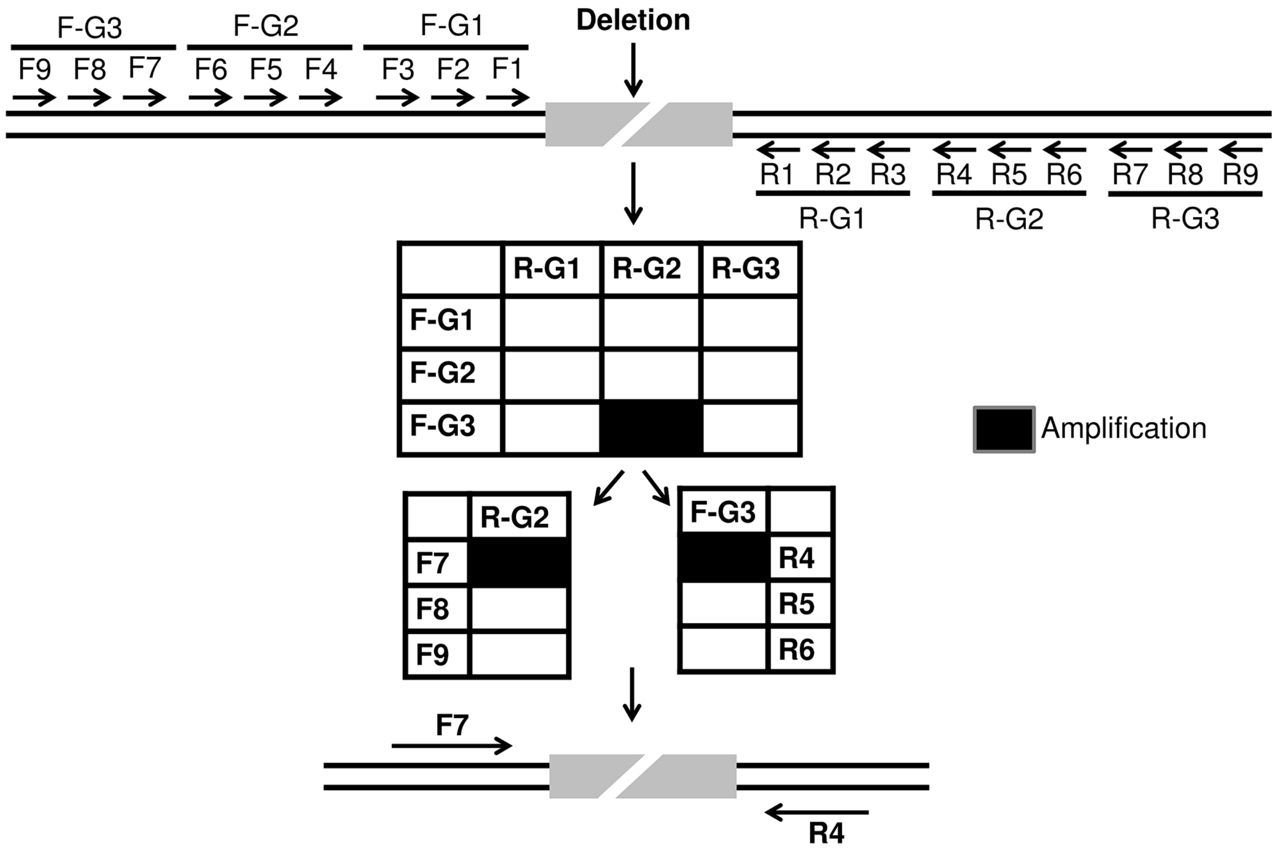

Supplementary Figure S5: A schematic representation of PAMP methodology. The deletion locus was flanked by nine primer pairs that were in turn grouped to contain three primers each. The forward and reverse primer groups were mixed in all possible combinations to result in 9 multiplex reactions. The positive amplicon (gray colored box) diluted 1:100 in water was used as template in repeat PCR using corresponding forward primer group and individual primers from the respective reverse primer group. In parallel, similar reaction was set using reverse primer group and individual primers from respective forward primer group. Finally, the genomic DNA was amplified using specific primers identified from repeat multiplex PCR.

Supplementary Table S1: Comparison of Chr 9p21 deletion coordinates in 5 cell lines analyzed (NCBI Build 37/hg19)

Cell line	Method	Telomeric side		Centromeric side		Deletion length (PAMP/IPCR)
		SNP CN≥1	SNP CN=0	SNP CN=0	SNP CN≥1	
MaMel-30	aCGH	rs7865071 (21,890,326)	rs10811638 (21,923,279)	rs17694493 (22,041,998)	rs10965224 (22,067,276)	141 kb deletion
	PCR validation	rs7865071 (21,890,326)	rs10811638 (21,923,279)	rs564398 (22,029,547)	rs17694493 (22,041,998)	
	PAMP	21,893,332		22,034,193		
MaMel-95	aCGH	rs7865071 (21,890,326)	rs10811638 (21,923,279)	rs10965224 (22,067,276)	rs16905599 (22,069,144)	181 kb deletion
	PCR validation	rs7039105 (21,855,096)	rs16938577 (21,860,428)	rs564398 (22,029,547)	rs17694493 (22,041,998)	
	PAMP	21,855,742		22,036,449		
MaMel-103a	aCGH	rs7846749 (21,780,251)	rs9298826 (21,787,848)	rs17799860 (24,709,463)	rs16908747 (24,717,136)	2,994 Mb deletion and 97 bp insertion (complementary strand of Chr9:24,718,771- 24,718,675)
	PCR validation	rs6475555 (21,716,997)	rs4341236 (21,727,208)	rs16908747 (24,717,136)	rs7854810 (24,725,322)	
	PAMP	21,725,260		24,718,843		
MaMel-19	aCGH	rs10119803 (21,666,066)	rs16938524 (21,673,153)	rs3731213 (21,986,218)	rs3731194 (21,991,752)	320 kb deletion and 1.1 Mb inversion (chr9:21,994,653-23,090,311)
	PCR validation	rs16938527 (21,673,608)	rs7866337 (21,679,452)	rs3731194 (21,991,752)	rs3218003 (22,000,770)	
	Inverse PCR	21,674,438		21,994,653		
MaMel-08a	aCGH	rs4468020 (20,996,709)	rs1857647 (21,016,929)	rs41370049 (24,353,310)	rs16908321 (24,375,619)	3,183 Mb deletion and translocation (telomeric side: t(9;15)(p21.3;-q12); centromeric side t(9;6)(p21.3; p12.1))
	PCR validation	rs10757178 (21,046,674)	rs2383177 (21,068,841)	rs17197000 (24,244,133)	rs17197195 (24,247,485)	
	Inverse PCR	21,062,696		24,246,191		

aCGH: array comparative genomic hybridization, SNP: single nucleotide polymorphism, CN: Copy number, PAMP: primer approximation multiplex PCR.

Supplementary Table S2: Sequence of characterized breakpoint junctions in 5 melanoma cell lines(NCBI Build 37/hg19).

See Supplementary File 1

Supplementary Table S3: Primers used in the analysis

Cell lines	Forward primer	Reverse primer
**For cloning breakpoints**		
MaMel-30	GAACCACATCTTGACCATAAAGC	TCCCAGGATATCTCACCAAAGTA
MaMel-95	GAAGGGCATGATTGAGTAGAGTG	CAAACTGGTACATTGATGTCAGG
MaMel-103a	ATATGTCCCAGGCAAAAGATACA	AACCCTTCTACACATTGCTTTAGG
MaMel-19: Breakpoint (telomeric)	CTAGGATGGAGACAGGCAGATTA	CGATAAACCAGAAATGAATGAGG
MaMel-19: Breakpoint (centromeric)	GCTGTTCCTGGTGATTAACTGCT	AGATGACCTCGCTTTCCTTTCTT
MaMel-08a: Breakpoint (telomeric)	GAAGCCTCCTAAATCCCAATCT	TGCCTAGGCTTGTGTTAATTTC
MaMel-08a: Breakpoint (centromeric)	CAATGGTTATGGACCTTGTTATT	ATCCCATTGTCATCTCAATACTT
**For inverse PCR**		
MaMel-19 (telomeric)	AACTGTGATGGCAGCTTGTCTA	ATCATTCAATTCCTTCCCATTG
MaMel-19 (centromeric)	CTCGCGTAGAATGGTTGTCTTG	AAGAGGAAGAAGCGCTCAGATG
MaMel-08a (telomeric)	AAGAGCGCTTTCAAGTACAGCTT	AGGGCAATCATGAGTTAAGACCT
MaMel-08a (centromeric)	CAGGATGGACTACAATTCTGAGC	AGTTCATCATCCCATTGTCATCT
**For RACE**		
MTAP-EX4-F	ATGCCTTCAAAGGTCAACTA	
F65	AAGGAGCACGAGGAAGCATGTCC	
F75	GAAACCCTCCATAACCTGAAGTGTCC	
**For cloning into pET20b**		
Fwd-primer_NdeI	GTCA***CATATG***GCCTCTGGCACCACC	
**For reverse transcription PCR**		
MTAP-EX4-F	ATGCCTTCAAAGGTCAACTA	
ANRIL-EX6-R		CTGTTACCTCTGATGGTTTCTT
MTAP-EX4-F2	AGGCGAACATCTGGGCTTTGA	
ANRIL-EX6-R2		CAGTACTGACTCGGGAAAGGA
**For the whole transcript**		
MTAP-EX1-F	GCTCTGTGGCTCGCTTGGTT	
MaMel-30-transcript1_2		TGCTGTTAAAAGTTGGCCCATG
MaMel-95-transcript1		TTTTTGCTAAACCATCTCTTTTTCTG
